# The dominant role of climate change in determining changes in evapotranspiration in Xinjiang, China from 2001 to 2012

**DOI:** 10.1371/journal.pone.0183071

**Published:** 2017-08-25

**Authors:** Xiuliang Yuan, Jie Bai, Longhui Li, Alishir Kurban, Philippe De Maeyer

**Affiliations:** 1 State Key Laboratory of Desert and Oasis Ecology, Xinjiang Institute of Ecology and Geography, Chinese Academy of Sciences, Urumqi, China; 2 University of Chinese Academy of Sciences, Beijing, China; 3 Department of Geography, Ghent University, Ghent, Belgium; 4 Sino-Belgian Joint Laboratory of Geo-information, Urumqi, China and Ghent, Belgium; Tennessee State University, UNITED STATES

## Abstract

The Xinjiang Uyghur Autonomous Region of China has experienced significant land cover and climate change since the beginning of the 21^st^ century. However, a reasonable simulation of evapotranspiration (ET) and its response to environmental factors are still unclear. For this study, to simulate ET and its response to climate and land cover change in Xinjiang, China from 2001 to 2012, we used the Common Land Model (CoLM) by adding irrigation effects for cropland and modifying root distributions and the root water uptake process for shrubland. Our results indicate that mean annual ET from 2001 to 2012 was 131.22 (±21.78) mm/year and demonstrated no significant trend (*p* = 0.12). The model simulation also indicates that climate change was capable of explaining 99% of inter-annual ET variability; land cover change only explained 1%. Land cover change caused by the expansion of croplands increased annual ET by 1.11 mm while climate change, mainly resulting from both decreased temperature and precipitation, reduced ET by 21.90 mm. Our results imply that climate change plays a dominant role in determining changes in ET, and also highlight the need for appropriate land-use strategies for managing water sources in dryland ecosystems within Xinjiang.

## Introduction

Due to global warming and human activities, acceleration and intensification of the global water cycle is becoming an undisputed fact [1, 2]. Evapotranspiration (ET), an important component of hydrological processes, plays a critical role in the global water budget. Oki and Kanae [3] reported that global land ET returns approximately 60% of annual land precipitation to the atmosphere, indicating that asynchronous changes in ET may lead to severe hydrological deficits [4]. Additionally, ET consumes more than half of solar radiation in the form of latent heat [5]. Shukla and Mintz [6] indicated that the Northern Hemisphere would be 15°C– 25°C warmer if land ET was assumed to be zero, suggesting important implications for regional climate variability. From 1982 to 1997, global annual ET increased at an average rate of 0.71 mm/year but stalled and even decreased coincident with the last major El Niña event of 1998, with the decreasing trend continuing until 2008 [7]. However, Zhang et al. [4] reported that reduced ET growth between 1998 and 2008 was an episodic phenomenon, with subsequent recovery for the ET growth rate following 2008. Although efforts have been made to investigate ET variability on a global scale, a comprehensive analysis of ET changes and their response to environment factors (e.g., climate variability and land cover change (LCC)) are still lacking on a regional scale.

Local scale measurements and observations of ET have been widely conducted within various ecosystems [8]. However, dense global or regional coverage ET cannot be represented using field measurements. Satellite- and statistics-based technologies have provided an alternative method for estimating ET on global and regional scales [7, 9] although neither of these methods can qualify the relative roles of environmental factors in controlling ET. Land surface models (LSMs) provide a possible resolution for simulating ET and quantifying the contributions of various environmental factors on ET. However, most global LSMs underestimate ET within arid/semi-arid regions [10–12]. A possible reason for this phenomenon is that most current LSMs cannot capture special vegetation structures and their related functioning. When soil water is limiting, most species within dryland ecosystems do not suffer from water stress [12]. Several adaptive mechanisms can account for this finding. Being challenged with climates with extreme aridity and heat conditions, species have evolved into rich and deep root distributions with high root/shoot ratios [13] and these specific morphological root distributions can influence ET through impacts on water uptake. Additionally, Zheng and Wang [12] reported that some stressed portions of the root zone do not necessarily indicate the water stress of entire plants. As a result, reduced water uptake from the stressed portion of the root zone can be compensated for by enhanced water uptake over portions of the root zone where water is more available. Furthermore, hydraulic redistribution (HR) is a widespread phenomenon where plant roots take-up water from deep moist soils and redeposit it into the upper soil layer at night for the purpose of root uptake to sustain daytime transpiration [14, 15]. Although the above processes play a vital role in influencing ET within arid/semi-arid regions, few LSMs have taken conjointly into consideration when simulating ET.

The Xinjiang Uyghur Autonomous Region of China is located deep inside the Eurasian continent and is characterized by extreme low precipitation and high temperatures. The region is unique and, over the years, has experienced a dynamic land-use history and climate change [16]. Hu et al. [17] reported that annual mean temperatures for this region have increased at an average rate of 0.39°C/decade from 1979 to 2011. Additionally, from 1982 to 2011, precipitation within this region has also experienced an increase at a rate of 8 mm/decade [18]. However, this dramatic rise in both temperature and precipitation stalled and even turned into decrease since the 21^st^ century [19]. With a constant increase in population and accelerating industrial development and urbanization, oases (as a primary site for human settlement due to the availability of fertile soils and fresh runoff from surrounding mountains) have significantly expanded within this region [20]. Following the expansion of oases, local native vegetation was modified by irrigation and cultivation, as well as reclamation, and most native vegetation has been changed into cropland. Previous study has concluded that climate change plays a dominant role in controlling ET variability over large regions, whereas land-use changes have dramatically affected ET within watersheds with significant land conversion [21]. Nevertheless, knowledge gaps still exist for understanding ET in response to changing climate and land cover in this typical arid/semi-arid region.

For this study, we used a process-based land surface model, the Common Land Model (CoLM), to simulate ET in Xinjiang Uyghur Autonomous Region of China from 2001–2012. Major objectives of the study were the following: (1) improve the performance of the CoLM for simulating ET (shrubland and cropland); (2) restructure the temporal and spatial distribution of ET; and (3) investigate the relative contributions of climate change and LCC on ET variability.

## Materials and methods

### The study area

**Fig 1:**
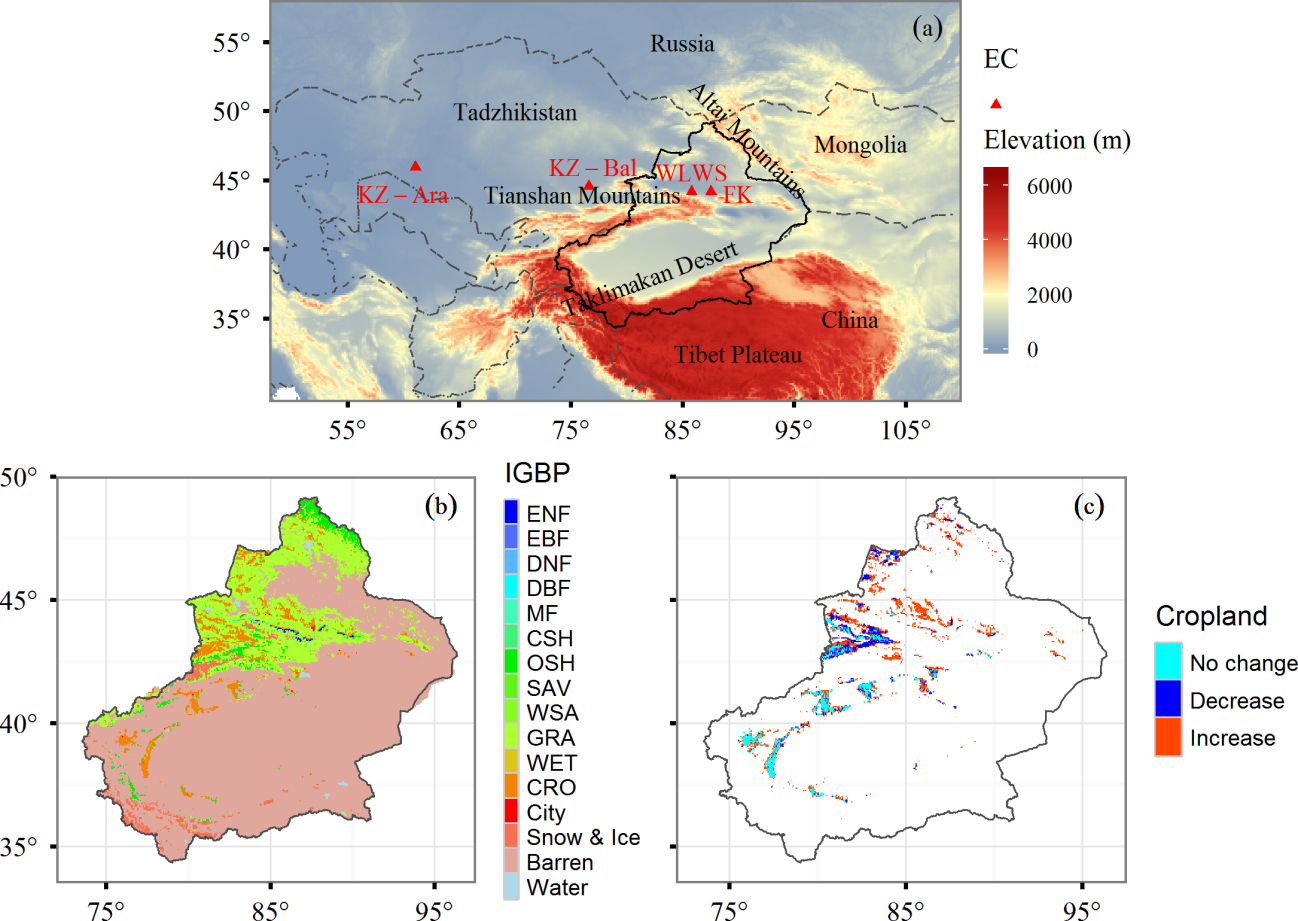
**The study area and the distribution of the flux tower sites (a), the vegetation types (b), and the spatiotemporal changes of cropland from 2001 to 2012 (c).** EC: Eddy covariance flux tower station. FK: Fukang station; KZ-Ara: Aral Sea station in Kazakhstan; KZ-Bal: Balkhash Lake station in Kazakhstan; WLWS: Wulanwusu station. ENF: Evergreen needleleaf forest; EBF: Evergreen broadleaf forest; DBF: Deciduous broadleaf forest; DNF: Deciduous needleleaf forest; MF: Mixed forest; CSH: Closed shrub; OSH: Opened shrub; SAV: Savanna; WSA: Woody savanna; GRA: Grassland; WET: Wetland; CRO: Cropland.

Xinjiang Uyghur Autonomous Region of China (34°N-49°N, 73°E-96°E) is one of the driest regions in the world. Approximately 73% of the land area (1.66 M km^2^) is covered by deserts, while a lower percentage (~9%) is covered by desert vegetation primarily dominated by xeric shrubs (e.g., *Haloxylon ammodendron* and *Tamarix ramosissima*) [22]. Only ~1% of the land is covered by forests that are largely distributed near mountainous areas (Fig 1). Since the onset of the 21^st^ century, Xinjiang has undergone dramatic land-use changes and cropland increased by 11% from 2001 to 2012. During the period from the 1980s to 1990s, Xinjiang experienced a significant warming and increasing wet trend [23]. However, precipitation has diminished and warming trends have stalled and even reversed since the onset of the 21^st^ century [19]. Mean annual precipitation is lower than 200 mm/year yet displays divergent spatial patterns of less than 50 mm/year for the Taklimakan Desert and more than 400 mm/year for the Tianshan Mountains. Mean annual temperatures vary from ~3°C within the Tianshan Mountains to ~13°C within the Taklimakan Desert.

### The common land model

The CoLM used in this study is a process-based land surface model that allows simulations of biogeochemical and biophysical mechanisms, including representations of water, carbon, and energy processes [24]. The initial version of the model (Community Land Model (CLM)) was proposed in order to provide a framework for a true community developed land component for the National Center for Atmospheric Research (NCAR) Community Climate System Model (CCSM), and has been updated and developed by open scientific collaboration. The last version of the model (CoLM) used a one-layered, two-big-leaf sub-model for photosynthesis, stomatal conductance, leaf temperature, and energy fluxes instead of the big-leaf treatment of the CLM for overcoming overestimations of CO_2_ and water vapor [25]. Model performance has been validated on a global scale over time using extensive field data for various biomes. Like most other land surface models, CoLM builds on the concept of plant functional types to describe vegetation attributes. CoLM handles the effects of LCC by changing spatially explicit LCC datasets. However, underestimations of water fluxes still exist because the model cannot capture the form and structure of desert plants and related root functions. Besides, it also cannot account for irrigation effects on ET for cropland. We provide a brief description of these processes below, in order to discuss the CoLM modifications we employed for cropland and shrubland.

### The irrigation effects for cropland

Water is the most limiting resource for sustaining crop production in agricultural oases in Xinjiang, and almost 90 percent of agricultural water was used for irrigation [26]. Therefore, all the cropland was treated to be irrigated in our study area to account for irrigation effects on ET. We hypothesized that the irrigation was activated when available water in the topsoil layers with the depth of 0.3 m decreases to a threshold of the maximum available water (field capacity minus wilting point) during the growing season. After the irrigation, the soil moisture will reach the field capacity. This method has been widely applied in other studies [27, 28]. The flux tower observations from Wulanwusu (WLWS) Agro-meteorology Experimental Station were used to calibrate the threshold, which was experimentally determined as 0.27 in this study.

### The root distribution function

For this study, the nonlinear model used for interpolations of root distributions was a logistic dose-response curve [29] that was fitted to the following cumulative root profiles:

$$r(D)=\frac{R_{max}}{1+{\left(\frac{D}{D_{50}}\right)}^{c}}$$ (1)

$$c=\frac{-1.27875}{{log}_{10}D_{95}-{log}_{10}D_{50}}$$ (2)

where r(D) is the cumulative amount of roots above the soil profile depth (D); R_max_ is the root quantity, set as 100%; and D_50_ and D_95_ are the depths to which root quantities are equal to 50% and 95% of all roots, respectively. We modified the D_50_ and D_95_ default values of 170 cm and 240 cm to 47 cm and 302 cm according to our observed vertical root distribution for a typical desert plant (*Tamarix*) [10].

### The root water uptake function

In the default CoLM, the Root Water Uptake Function (RWUF) is given by the following:

$$\varepsilon_{j}=\frac{f_{root,j}a_{j}}{\sum f_{root,j}a_{j}}$$ (3)

$$a_{j}={\left(\frac{\varphi_{wilt-}\varphi_{j}}{\varphi_{wilt+}\varphi_{sat}}\right)}^{m}$$ (4)

where ε_j_ and f_root,j_ are the root water uptake fraction and the root distribution fraction within the jth soil layer, respectively; φ_wilt_ is the wilting point potential; φ_sat_ is the soil water matric potential at the saturation point; φ_j_ is the soil water matric potential within the jth soil layer; a_j_ is the root water uptake efficiency within the jth soil layer; and m is an empirical coefficient. When the value of m is equal to one, a_j_ is the same as the default value provided by the CoLM. Li et al. [11] determined that when m is set lower than one the new root water uptake efficiency always computes values that are larger than the default. The soil water uptake efficiency with the new a_j_ was thus higher than with a default a_j_, especially for low soil water conditions (the low soil water matrix potential). This procedure is appropriate for an assumption that desert plants maintain their physiological activities under low matrix potentials [13]. For this study, the value of parameter m was empirically determined to be 0.01 according to Li et al. [11].

### The hydraulic redistribution function

Water flux amongst various soil layers due to HR through the root conduit was followed by Ryel et al. [15] and Lee et al. [30], which can be formulated as:

$$H_{j}=C_{RT}\sum_{j=1}^{n}\left(\varphi_{j}-\varphi_{i}\right)max({\text{c}}_{\text{i}},c_{j})\frac{f_{root,i}f_{root,j}}{1-f_{root,x}}\delta_{T}$$ (5)

where C_RT_ is the maximum radial soil-root conductance of the entire active root system with a constant value of 0.097 (cm/Mpa/h) [15]; n is the number of soil layers; φ_i_ and φ_j_ are the soil water potential within the ith and jth soil layer, respectively; f_root,i_ and f_root,j_ are the root fraction within the ith and jth soil layer, respectively; f_root,x_ = f_root,j_ when the soil water content within the j layer is larger than it is within the i layer, or f_root,x_ = f_root,i_ otherwise; and δ_T_ is a switch controlling HR, which is generally only valid at night, that is equal to 0 during the day and 1 at night. The relative soil-root conductance for water within ith (c_i_) or jth (c_j_) soil layer, is calculated using the following empirical relationship:

$${\text{c}}_{\text{i}}=\frac{1}{{1+(\frac{\varphi_{i}}{\varphi_{50}})}^{b}}$$ (6)

where φ_50_ is the soil water potential when the soil hydraulic conductance is reduced by 50%, and b is an empirical constant.

### The data source

The 3-hour climate dataset from 1996 to 2012 at one degree was obtained from Princeton University and includes temperature, precipitation, downward short- and long-wave radiation, specific humidity, surface air pressure, and wind speed [31]. The Princeton dataset is one of the most widely used reanalysis climate datasets for driving land surface models. The vegetation distribution map from 2001 to 2012 was derived from MODIS (Moderate Resolution Imaging Spectroradiometer) land cover type products (MCD12C1.005) at a spatial resolution of 0.05 degree. The classification method was performed according to the International Geosphere-Biosphere Program (IGBP). Other input datasets included soil texture, soil color, and a topographic map (elevation) from the United States Geological Survey (USGS) used to define land surface characteristics.

To evaluate the performance of the CoLM in simulating ET, four flux tower observations at the site level were used to validate our model simulation. Detailed descriptions of the flux towers are provided in Table 1. Two ET datasets were used to compare our simulations at a regional level. One was produced by the Penman–Monteith equation driven by MODIS data and daily meteorological data (MOD16A3) [9], and the other was produced by a Machine-Learning Algorithm (MLA) by combining flux-tower ET measurements and remote sensing observations [7].

**Table 1:** The name, location, vegetation type, annual mean temperature (AMT), annual precipitation (AP), and time period of observations used for model calibration and validation.

Site	Longitude	Latitude	Vegetation type	AMT	AP	Time period
FK	87°56´E	44°17´N	Shurb	6.6°C	163 mm	2007–2009
KZ-Bal	76°39´E	44°34´N	Grass	5.7°C	140 mm	23 May—6 September 2012
KZ-Ara	61°05´E	45°58´N	Shrub	8.3°C	140 mm	30 April—18 August 2012
WLWS	85°49´E	44°17´N	Crop	7.0°C	210 mm	2009–2010

FK: Fukang station; KZ-Ara: Aral Sea station in Kazakhstan; KZ-Bal: Balkhash Lake station in Kazakhstan; WLWS: Wulanwusu station.

### The experimental design

First, two simulations were designed to assess the irrigation effects for cropland at WLWS station. One simulation was executed with adding irrigation effects and the other without adding irrigation effects. And then the estimated ET of above two simulations was compared to observations. Second, we ran four simulations to validate the performance of improved CoLM in shrubland at FK station. The first simulation runs with adding HR; the second with modifying RWUF; the third with both adding HR and modifying RWUF; the last with default runs. And then, all the outputs of above simulations were compared to observations. Finally, six simulations were designed to quantify the relative contributions of the two factors (LCC and climate change) to ET variability (Table 2). One simulation was termed “LCC_CLIM” and was designed to assess the combined effects of climate and land cover change on ET from 2001 to 2012. Other simulations were designed for assessing ET variability on individual factors or a subset of environmental factors. “LCC”, “PREC”, and “TEMP” simulated the individual effects of land cover change, precipitation, and temperature, respectively. “CLIM” simulated the combined effects of all climate factors (e.g., precipitation, temperature, radiation). “COMB” simulated the combined effects of both temperature and precipitation. In the factorial analyses, only the factor(s) under investigation varied over time while other environmental drivers were kept at the 2001 initial level. To prevent abnormal fluctuations, an equilibrium run was designed prior to each simulation in order to make the natural ecosystem reach an equilibrium state. For this step, the model was driven by the initial climate state in 1996.

**Table 2:** The design of the simulation experiments.

Experiment	LCC^b^	Climate		
		Temperature	Precipitation	Others
LCC_CLIM^a^	2001–2012	2001–2012	2001–2012	2001–2012
LCC^b^	2001–2012	2001	2001	2001
CLIM^c^	2001	2001–2012	2001–2012	2001–2012
PREC^d^	2001	2001	2001–2012	2001
TEMP^e^	2001	2001–2012	2001	2001
COMB^f^	2001	2001–2012	2001–2012	2001

^a^LCC_CLIM, combined land cover change and climate change

^b^LCC, land cover change

^c^CLIM, climate change

^d^PREC, precipitation

^e^TEMP, temperature

^f^COMB, combined temperature and precipitation

### The statistical analysis

A linear fitting method was used to compute the trend of ET and climatic variables. A mean value difference between two individual periods was used to quantify the temporal changes of ET, and the two periods are defined as the last and first three years during 2001–2012. The coefficient of determination (*R*^2^) and the Root Mean Square Error (*RMSE*) were used to evaluate agreement between simulations and observations. The *t*-test was used to assess the statistical significance (*p*) of the trend and the *R*^2^. *R*^2^ and *RMSE* were calculated as follows:

$$R^{2}=\frac{\sum {\left({ŷ}_{i}-\overset{-}{y}\right)}^{2}}{\sum {\left(y_{i}-\overset{-}{y}\right)}^{2}}$$ (7)

$$RMSE=\sqrt{\frac{\sum {\left({ŷ}_{i}-y_{i}\right)}^{2}}{n-1}}$$ (8)

where y_i_, ${ŷ}_{i}$, and $\overset{-}{y}$ indicate the observed, modeled, and mean of the observed ET at time step i, respectively, and n is the number of observations.

## Results

### The model performance evaluation

**Fig 2:**
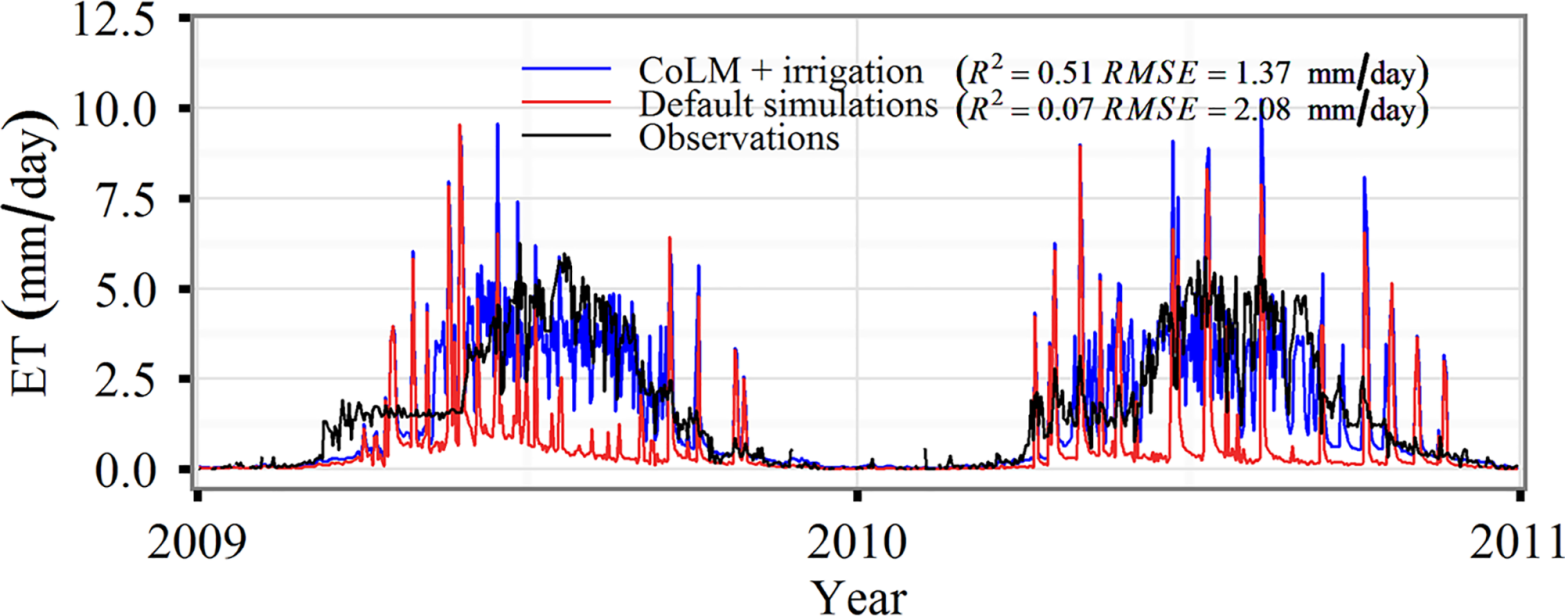
A comparison of evapotranspiration (ET) between simulations and observations. Daily averaged ET at the Wulanwusu (WLWS) site based on the default common land model (CoLM), and the modified CoLM with the addition of irrigation effects (CoLM + irrigation).

Fig 2 shows the performance of CoLM in simulating ET with and without adding the irrigation effects at WLWS Agro-meteorology station. The default CoLM without adding the irrigation effects underestimated ET especially in the growing season, while the modified the CoLM under considering the irrigation effects improved the accuracy of simulations. The *R*^2^ increased from 0.07 to 0.51 and *RMSE* reduced from 2.08 to 1.37 mm/day.

**Fig 3:**
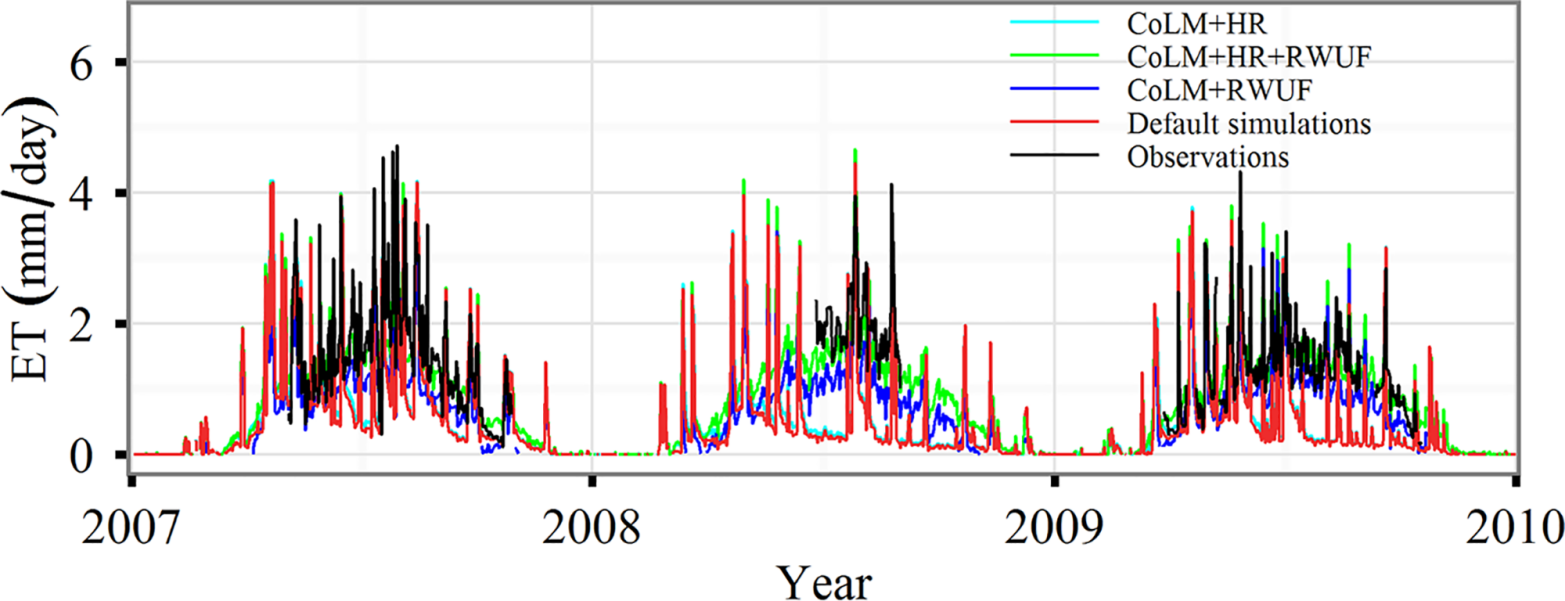
A comparison of evapotranspiration (ET) between simulations and observations. Daily averaged ET at the Fukang (FK) site based on the default common land model (CoLM), the modified CoLM with the addition of Hydraulic Redistribution (HR) (CoLM+HR), the modified root water uptake efficiency (RWUF) (CoLM+RWUF), their combination (CoLM+HR+RWUF), and the observations.

Our results indicated that modified CoLM simulations were more comparable to observations at the FK (Fukang) site than the default CoLM. Fig 3 shows that CoLM-estimated ET had a higher performance in catching seasonal patterns by adding HR and modifying RWUF. A high ET occurred during the growing season and a low ET occurred during the winter. However, ET was underestimated especially during the growing season by using the default CoLM. The CoLM modified using both the HR and RWUF produced a more significant linear relationship than that modified by HR, with *R*^2^ = 0.72, *p* <0.05, and *RMSE* = 1.04 mm/day (Table 3). However, the CoLM modified using both the HR and RWUF showed a comparable role with that only by modifying RWUF. The CoLM modified by adding HR showed marginally better performance than default CoLM. The *R*^2^ increased from 0.43 to 0.45, and *RMSE* decreased from 1.66 to 1.61 mm/day.

**Table 3:** Model performance for simulating ET as indicated by the coefficient of determination (*R*^2^) and the root mean square error (*RMSE*, mm/day).

Site	Default simulation		CoLM+HR		CoLM+RWUF		CoLM+HR+RWUF	
	*R*^2^	*RMSE*	*R*^2^	*RMSE*	*R*^2^	*RMSE*	*R*^2^	*RMSE*
FK	0.43*	1.66	0.45*	1.61	0.72*	1.04	0.72*	1.04
KZ-Bal	0.90*	2.71	0.90*	2.71	0.90*	2.71	0.90*	2.71
KZ-Ara	0.36*	1.44	0.36*	1.44	0.37*	1.42	0.37*	1.42

CoLM+HR, CoLM+RWUF, and CoLM+HR+RWUF indicate that the default model CoLM was improved by adding Hydraulic Redistribution (HR), by modifying the root water uptake efficiency (RWUF), and by both (HR and RWUF) combined, respectively. *R*^2^ with an asterisk indicates significance (*p* < 0.05).

Because of a lack of situ observations, two flux tower observations, adjacent to our study area were also used to compare to our simulation. A strong linear relationship obtained from the CoLM modified using both the HR and RWUF was determined (*R*^2^ = 0.90, *p <*0.05) at the KZ-Bal site, while the relationship was moderate at the KZ-Ara site (*R*^2^ = 0.37, *p* <0.05) (Table 3). The *RMSE* was 2.71 and 1.42 mm/day at the KZ-Bal and KZ-Ara sites, respectively. The results imply that overall predictions for the modified CoLM matched well with field observations. The CoLM modified by either the HR or RWUF did not impact performance for simulating ET for grass types at the KZ-Bal site. Finally, the CoLM modified using both the HR and RWUF was selected for performing regional simulations.

**Fig 4:**
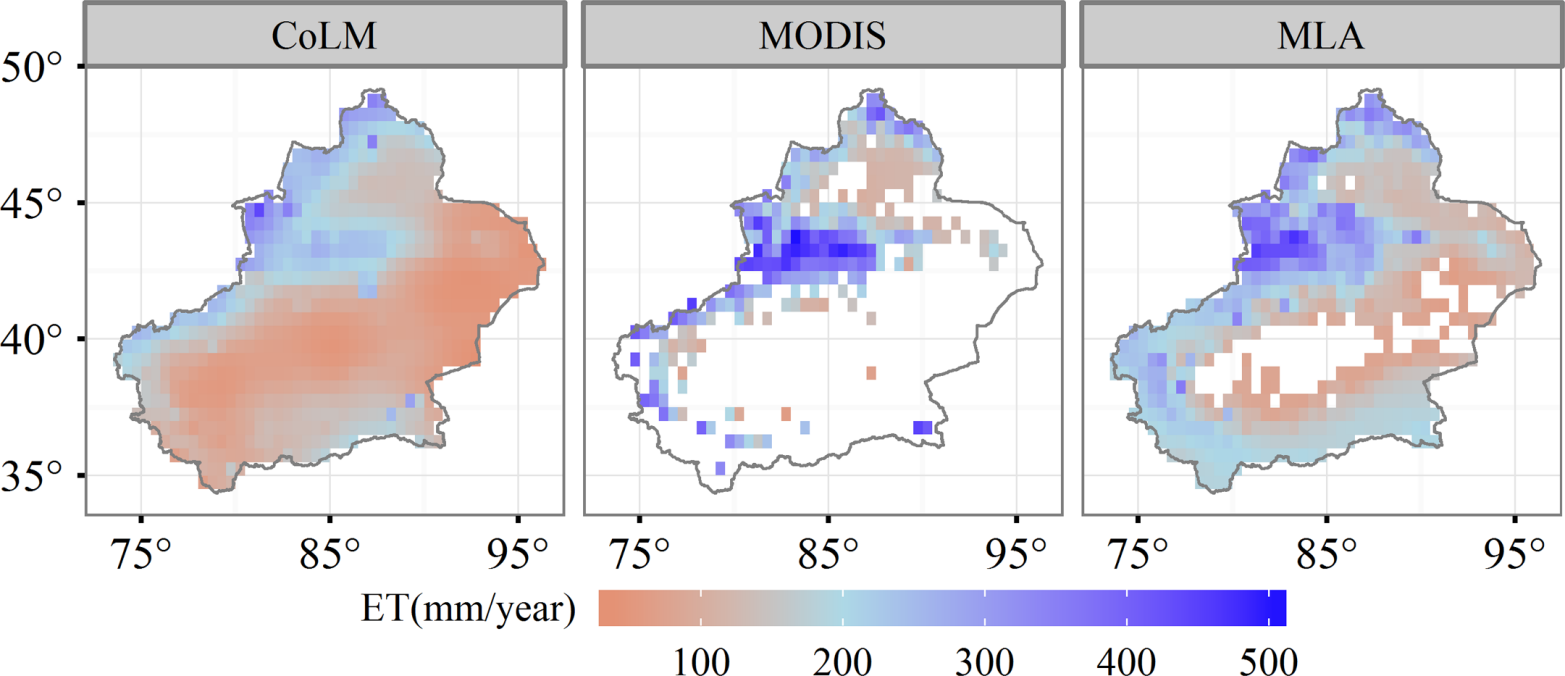
Spatial patterns of modified CoLM-simulated, MODIS-estimated, and MLA-simulated evapotranspiration.

We evaluated the spatial pattern of modified CoLM-simulated ET at the regional level by comparing it to the MODIS product (Fig 4). Average ET estimated using the CoLM agreed well with MODIS ET in terms of the spatial pattern from 2001–2012. High ET occurred in Tianshan and Altai mountainous areas, and low ET occurred in the Taklimakan Desert and adjacent areas. Our estimated ET was 131.22 (±21.78) mm /year, higher than the 91.17 (±5.85) mm/year derived from MODIS ET over the whole Xinjiang region. The discrepancy may result from a lack of values surrounding Taklimakan Desert areas for MODIS datasets. We further compared CoLM-estimated ET to MLA-estimated ET which is derived from Jung et al. [7]. Our estimates displayed a similar spatial pattern with MLA estimates, but a discrepancy in magnitude still existed. ET based on the MLA was ~200 mm/year for areas located in the south of our study area and was larger than our estimates of ~100 mm/year. Estimates for ET over Xinjiang were also less than MLA estimates, with values of 175.03 (±4.06) mm/year.

**Fig 5:**
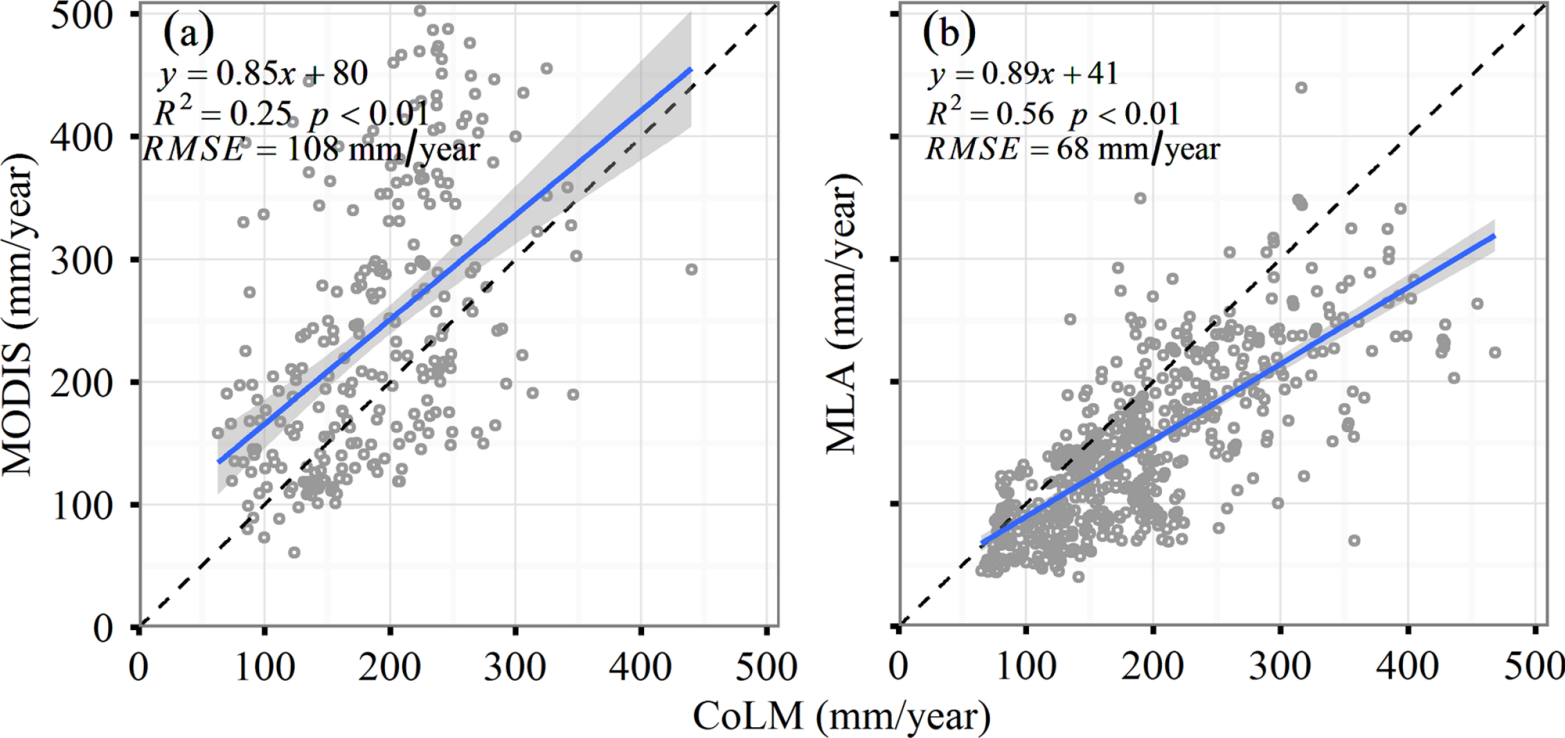
**Comparisons of CoLM-simulated versus MODIS-estimated (a) and MLA-simulated (b) evapotranspiration.** The blue line represents the least squares regression. The dashed line indicates the 1:1 line.

We further selected all the grids from MODIS-based and MLA-based ET to compare them to our simulations. The results showed a poor correlation between CoLM-estimated and MODIS-estimated ET (*R*^2^ = 0.25, Slope = 0.85, and *RMSE* = 108 mm/year), but a moderate correlation between MLA-estimated and CoLM-estimated ET (*R*^2^ = 0.56, Slope = 0.89, and *RMSE* = 68 mm/year) (Fig 5).

### Temporal and spatial changes in ET

**Fig 6:**
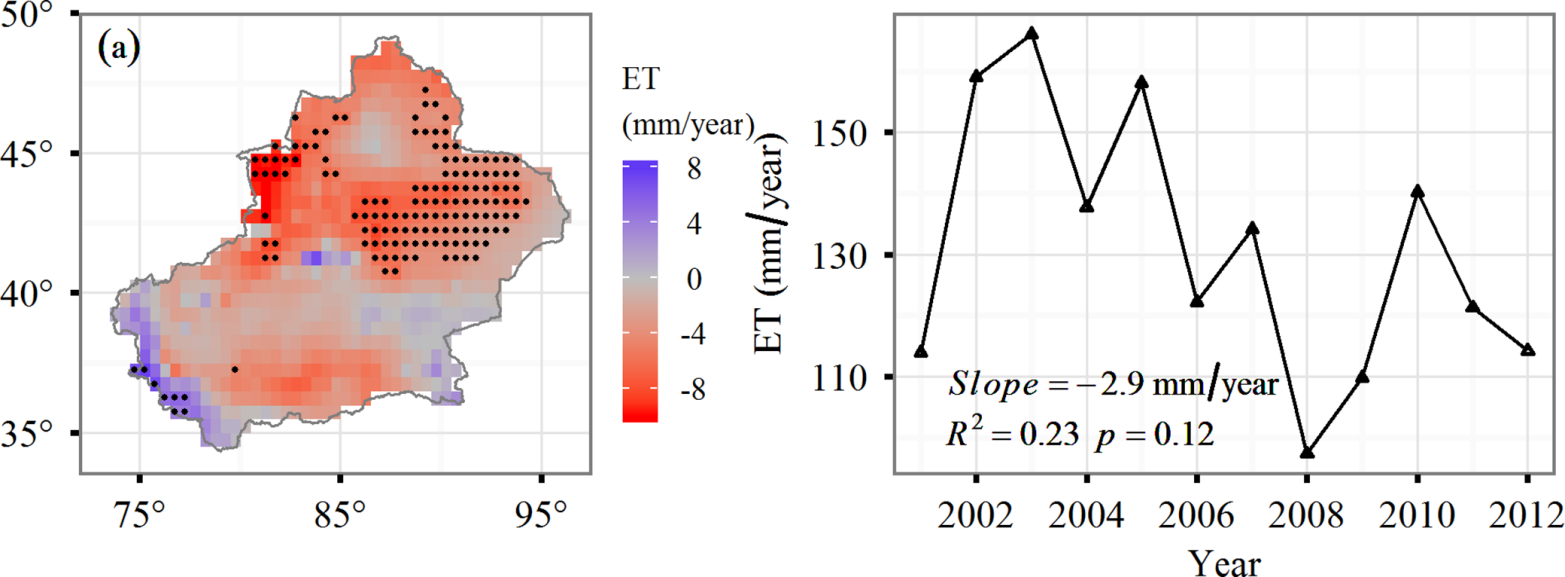
**The spatial (a) and temporal (b) variability of annual ET in Xinjiang from 2001–2012.** Pixels with black points are statistically significant at *p* <0.05. The blue solid line represents the least squares regression and the shadow indicates the confidence interval.

Fig 6 shows the temporal and spatial patterns of CoLM-estimated annual ET in Xinjiang Uyghur Autonomous Region of China. From 2001 to 2012, we estimated a mean annual regional land-surface ET value of 131.22 (±21.78) mm/year. Average ET in this region exhibited significant inter-annual variability with a maximum in ET (166.09 mm) in 2003 and a minimum (97.39 mm) in 2008. No significant trend was determined for our study period (*R*^2^ = 0.23, *p* = 0.12). The spatial distribution is provided in Fig 6(A). Our results illustrate substantial spatial variations in ET across the study area. Approximately 13.5% of the pixels in this region experienced an increase in ET and only about 1% of the pixels were significant in southern areas. Decreased ET largely occurred in east areas and about 19.7% of the pixels were significant.

### Contributions of climate and land cover changes to ET

**Fig 7:**
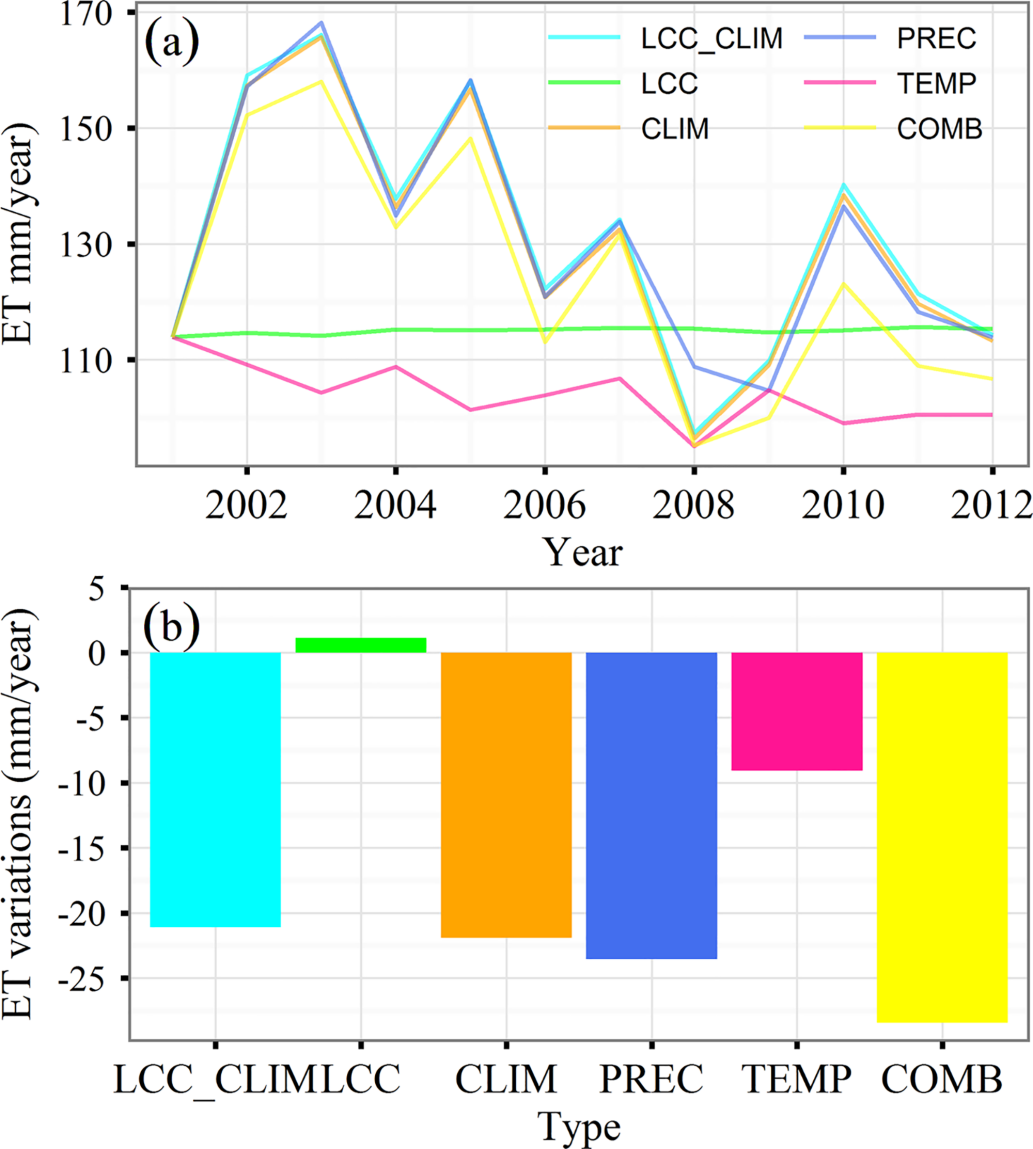
**Temporal variability of annual ET under different simulation scenarios (a) and contributions of environmental factors on evapotranspiration (ET) variability (b).** LCC_CLIM: Land cover change and climate change; LCC: Land cover change; CLIM: Climate change; PREC: Precipitation; TEMP: Temperature; COMB: Combined temperature and precipitation.

**Fig 8:**
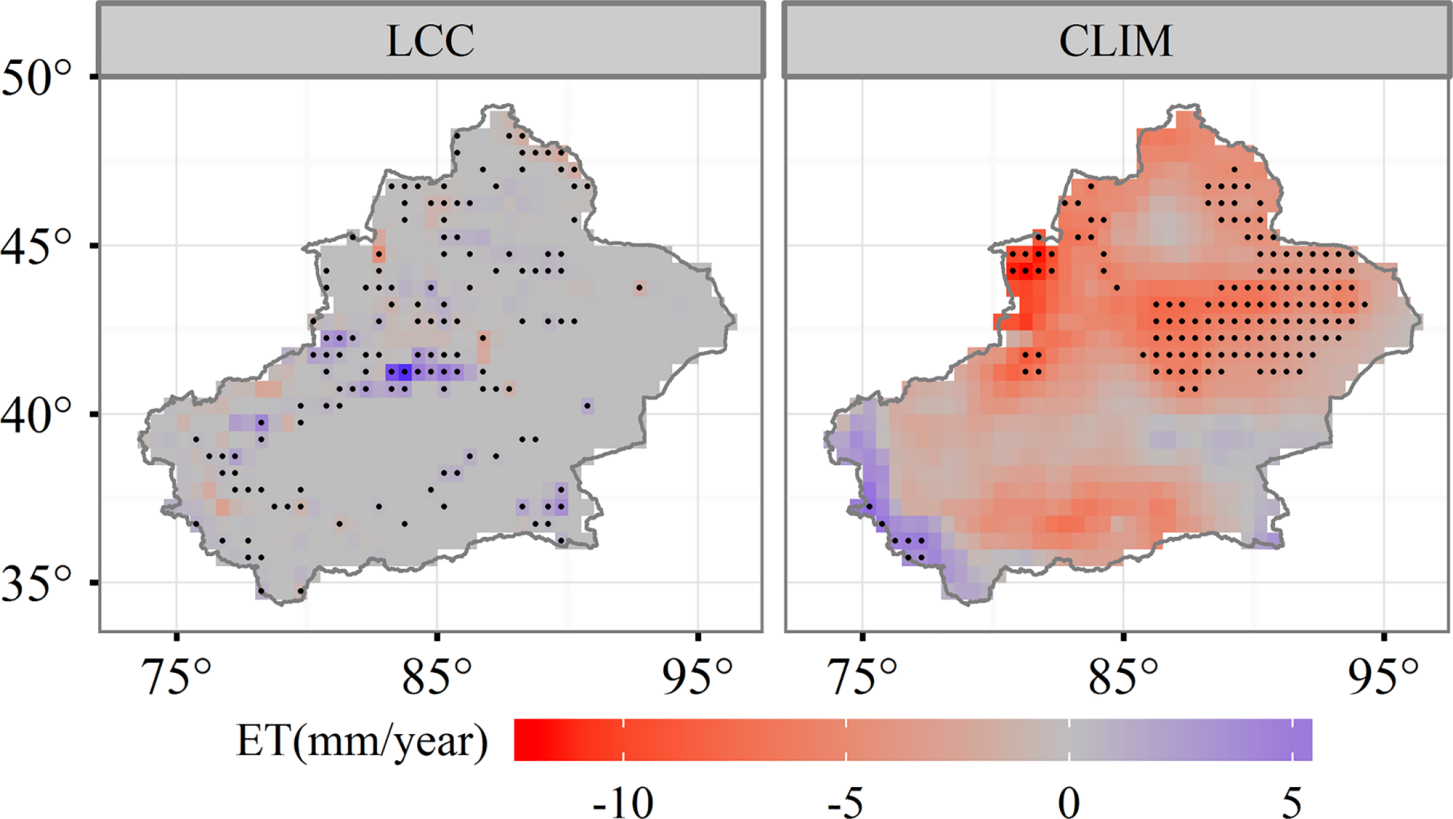
The effects of land cover change and climate change on evapotranspiration variability in Xinjiang.

Based on the results of scenario simulations, ET in response to LCC varied with time (Fig 7). The combined effect of both LCC and climate change reduced ET about 21.09 mm during the period from 2001 to 2012. Statistical analyses indicated that the LCC only accounted for 1% of the temporal variability of ET. The LCC simulation indicated that ET was enhanced by 1.11 mm from 2001 to 2012 (Fig 7(B)). More detailed information for LCC impacts on ET can be determined using the spatial distribution of ET variability based on the LCC scenario (Fig 8). Land conversion enhanced ET up to approximately 43.3% of pixels, and 13.8% of pixels were significant within southwestern Xinjiang and the northern Tianshan Mountains where the land cover type is primarily cropland (Fig 1(B)). Enhanced ET within the northern Tianshan Mountains may have resulted from an increase in croplands following 2001. Approximately 51.2% of pixels underwent a decrease in ET but only 6.2% of pixels, such as those in northern areas, were significant.

**Fig 9:**
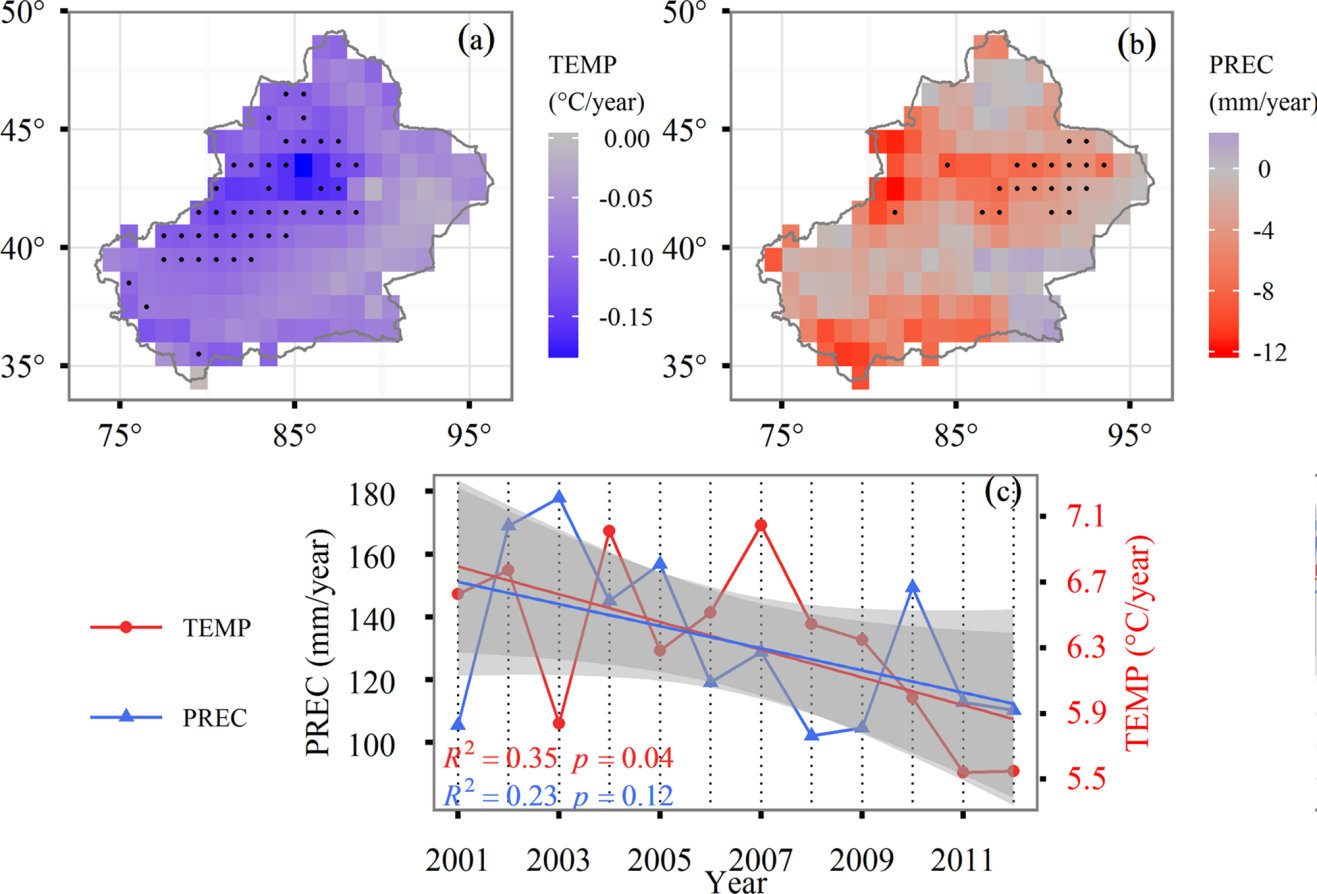
The temporal and spatial variability of annual mean temperature and precipitation in Xinjiang from 2001–2012. The spatial pattern of annual mean temperature (a), precipitation (b), and the temporal trend of annual mean temperature and precipitation (c). The solid line represents least squares regression and the shadow indicates the confidence interval.

Our model simulation suggested that climate effects accounted for 99% of the inter-annual variability of ET. During 2001–2012, the ET decreased with large fluctuations, especially during 2008 when ET decreased by approximately 17 mm (Fig 7). The CLIM simulation indicated that ET decreased by 21.90 mm from 2001 to 2012. Among climate factors, temperature change resulted in a 9.09 mm reduction and precipitation change resulted in a 23.55 mm reduction (Fig 7(B)). To illustrate the temporal correlation between ET and climate factors, we computed linear trends for temperature and precipitation from 2001 to 2012 (Fig 9). Our results indicated no significant trend for precipitation while temperature significantly decreased from 2001–2012 at a rate of 0.08°C/year. The temporal pattern of ET simulated using the CLIM scenario was coincident with precipitation. According to both PREC and TEMP simulations, the ET in 2008 was almost lowest (Fig 7(A)), suggesting that the dramatic decline in ET in 2008 may have resulted from a decrease in both precipitation and temperature.

Climate impacts on ET were negative over the majority of the study area. Approximately 88.3% of areas experienced a decrease in the CLIM-only scenario and only 20% of areas were significant. The spatial patterns of ET changes were very similar to precipitation. In the Tianshan Mountains the rate of decrease in ET exceeded 8 mm per year in some pixels and precipitation significantly decreased, indicating that the declining trend in ET was limited to the decrease of precipitation.

## Discussion

### A comparison of CoLM-simulated ET with previous studies

Assessments of ET variability and dynamics from the past to the future on either a global or regional scale have been comprehensively conducted [21, 32]. However, a lack of available field measurements (partially due to very limited or possibly biased samples) has made it difficult to gain a comprehensive view of ET estimates over the entire Xinjiang region. Based on the process-based CoLM, we estimated a mean annual ET of 131.22 (±21.78) mm/year for the period from 2001 to 2012, which is highly comparable with 130.64 mm/year estimated by Chen et al. [33] who used a remote sensing model (SEBS). However, in recent studies using a remote sensing method, Mu et al. [9] estimated a regional terrestrial ET of 91.17 (±5.85) mm/year, which is much lower than the CoLM-simulated terrestrial ET. A detailed comparison between the ET of the CoLM and other estimates is provided in Table 4. Recent studies have indicated several issues and uncertainties when retrieving ET using a remote sensing approach, and most of these uncertainties are related to surface variables such as land surface temperature, solar radiation, etc. [32]. Furthermore, the MODIS ET product from Mu et al. [9] does not cover sparse vegetation areas (i.e., deserts and the Gobi) due to limitations of retrieved vegetation index with negative values for dryland ecosystems, especially in Xinjiang.

**Table 4:** Comparisons of evapotranspiration between our study and past studies.

Methods	ET estimated (mm/year)	Time period	Reference
Process-based model (CoLM)	131.22 (±21.78)	2001–2012	This study
Remote sensing model (SEBS)	130.64	1980–2007	Chen et al (2013)
MODIS improved ET algorithm	91.17 (±5.85)	2001–2012	Mu et al (2011)
Machine learning approach	175.03 (±4.06)	2001–2011	Jung et al (2010)
Process-based model (CLM)	119.2 (±9.9)	1960–2005	Liu et al (2008)

Our estimated mean value of ET is lower than that of Jung et al. [7] who reported a regional terrestrial ET of 175.03 (±4.06) mm/year from 2001–2011. The ET estimation of Jung et al. [7] was upscaled from eddy covariance flux measurements to the global scale using a MLA method based on auxiliary remote sensing data. However, the method is a highly empirical mathematic model that does not consider environment factors and mechanisms. As reported, climate variability and LCC play a vital role in controlling ET changes [27]. In addition to these uncertainties, the MLA ET was largely based on flux tower measurements. However, flux towers in Xinjiang are sparse and highly uneven in distribution. In addition to these shortcomings, flux tower measurements themselves are often associated with an energy balance closure problem. All of the above-listed uncertainties could make the data less representative for the entire Xinjiang region.

The CoLM-estimated ET was larger than a regional terrestrial ET of 119.2 (±9.9) mm/year estimated by Liu et al. [34], who used another process-based model (CLM 2.0). The differences may come from different forcing datasets. Liu et al. [34] used global reanalysis forcing data from Qian et al. [35] and interpolated surface data based on meteorological observations in order to feed the CLM 2.0. We used global reanalysis data from Princeton [31]. Hu et al. [36] reported that reanalysis data sets contain large discrepancies for arid/semi-arid areas that have different accuracies in capturing terrain- and long-term drought-induced effects.

Additionally, current LSMs studies have a common problem with regard to underestimating ET in dryland ecosystems. Although deep root distribution and related root water uptake processes are important for the structure and function of dryland ecosystems, deep-root mechanisms are rarely considered in most land surface models [37]. In comparison, the improved CoLM in this study can address root distributions in deep soils based on field measurements. Besides, the process of RUWF is an important factor in controlling ET. Li et al.[11] has reported that linear RUWF in the default CoLM is the main reason that resulted in underestimating ET. An adjustment of RUWF from linear to nonlinear is expected to a reasonable solution to improve the performance of CoLM in simulating ET. In this study, the value of m in RUWF has been determined empirically from 1 to 0.01 based on the field measurements at FK station, which indicates that more soil water in modified CoLM will be absorbed than that in default CoLM. HR is also an important physical process contributing to dynamic root water uptake in plants that plant roots take-up water from deep moist soils and redeposit it into the upper soil layer at night for the purpose of root uptake to sustain daytime transpiration. This phenomenon has also been widely verified by previous studies in our study area [38, 39]. Therefore, all above modifications in CoLM could provide a more reasonable estimation of ET.

### The effects of climate factors and land cover changes on ET

We modeled the spatial and temporal pattern of ET changes and determined that the LCC contributed to the increase of regional ET. The increase in ET mainly occurred in regions where croplands were distributed. In these regions, the area of croplands underwent an increase of up to 11% that resulted in an upward trend for ET. Our results support previous conclusions that areas with significant land conversions dramatically affect ET on regional scales [21]. Luo et al. [40] reported that all increased cropland in Xinjiang resulted from dryland ecosystems such as shrublands, grasslands, and deserts that sustain their growth through irrigation. Due to irrigation and large leaf area indices, cropland has a higher ET than other land cover types. Tao et al. [41] reported that local human activities (such as irrigation) led to a decrease of the water volume diverted into the main stream of the Tarim River Basin in Xinjiang. The results of our study emphasized the significant impact of human activities on land-surface water sources, especially in arid/semi-arid regions where fresh available water largely results from runoff.

Our model simulation indicated that climate change and variability plays a negative role in controlling ET changes. Climate change reduced ET by 21.90 mm from 2001 to 2012. Among climate factors, temperature change resulted in a 9.09 mm reduction and precipitation change resulted in a 23.55 mm reduction. The combined effects of TEMP and PREC contributed to 28.45 mm reduction. Therefore, a large proportion of ET variability could not be explained by both precipitation and temperature alone. And other factors such as solar radiation and wind, etc. should be taken into consideration when interpreting climate change impacts on ET. According to our correlation analysis, the determined coefficient (*R*^2^) between ET and temperature (0.01) was less than that between ET and precipitation (0.94), indicating that precipitation has more influence on ET variability than temperature.

Climate change and variability directly impact the spatial distribution of ET changes. In the Tianshan Mountains, temperature decreased significantly and was associated with decreasing ET. As is known, temperatures in high elevation regions are generally low and limit vegetation activity. Such a finding is supported by multiple remote sensing studies that have determined that the Normalized Difference Vegetation Index (NDVI), an indicator of vegetation activity, in high altitudes is negatively correlated with temperature [42]. Decreased precipitation also contributed to the decreased ET that was largely determined over northeastern Xinjiang.

### Uncertainties and future research

Our modified model performance evaluation yielded a reasonable representation for ET estimates at the site level, and some differences may exist when compared to other surface ET products. The following uncertainties should be taken into consideration in interpretations of model estimates. As discussed above, the climate forcing data set is the primary factor controlling ET estimates, so assessments of different forcing data set impacts on ET estimates should be made and more accurate climate datasets at regional scales should be investigated in future research. Model structures and simplifications for some of the hydrological processes may also cause uncertainties. With the exception of climate and land cover change, we did not include the effects of other factors such as nitrogen deposition, ozone pollution, nitrogen fertilizer application, and elevated atmospheric carbon dioxide. Although former studies have indicated that nitrogen deposition and elevated carbon dioxide may not be as important as climate variability and land cover change on ET variability [27], they may impact ET dynamics over long-term periods.

## Conclusion

For this study, we provided a modified process-based land surface model (CoLM) for estimating ET in Xinjiang, China. Following modification by adding irrigation effects for cropland and modifying root distributions and the root water uptake process for shrubland, the CoLM was determined to provide high performance in simulating ET when compared to field measurements. The CoLM estimated a mean regional ET of 131.22 (±21.78) mm/year for Xinjiang from 2001–2012. Due to the relatively short time period, our estimate did not show a significant temporal trend for ET variability. The results of this study indicated that climate change plays a negative role in ET variability. Reduced precipitation, in conjunction with decreased temperature, reduced ET by approximately 28.45 mm. Our results also suggest that climate change played a dominant role in determining inter-annual variability in ET. Furthermore, cropland conversation was a critical factor for altering the spatial pattern of ET in Xinjiang. Our study highlights the need for taking appropriate strategies to manage water sources in Xinjiang, China.
